# Invasive Infections Caused by Streptococcus pseudoporcinus: A Two-Case Series and Review of the Literature

**DOI:** 10.7759/cureus.116454

**Published:** 2026-09-17

**Authors:** Julio de Jesús Navarro-Yépez, Frida Ximena Macías-López, David Alejandro Trejo-Osornio, Juan Manuel Gomez-Vasquez, Gilberto Gutierrez-Garcia

**Affiliations:** 1 Internal Medicine, Hospital General de San Juan del Río, San Juan del Río, MEX; 2 Education, Hospital General de San Juan del Río, San Juan del Río, MEX; 3 Pulmonology and Interventional Bronchoscopy, Hospital General de San Juan del Río, San Juan del Río, MEX; 4 Infectious Diseases Service, Hospital General de San Juan del Río, San Juan del Río, MEX; 5 Clinical Microbiology Laboratory, Hospital General de San Juan del Río, San Juan del Río, MEX

**Keywords:** chylous ascites, complicated parapneumonic effusion, invasive infections, spontaneous bacterial peritonitis, streptococcus pseudoporcinus

## Abstract

*Streptococcus pseudoporcinus* (*S. pseudoporcinus*)* *is a beta-hemolytic, Gram-positive, catalase-negative, nonmotile coccus that primarily colonizes the female genitourinary tract. Although it has historically been considered a colonizing organism, it has increasingly been associated with invasive infections. Its identification may be challenging because of its phenotypic similarity to other beta-hemolytic streptococci. We report two invasive infections caused by *S. pseudoporcinus*. The first occurred in a 59-year-old woman with cirrhosis who developed spontaneous bacterial peritonitis in the setting of chylous ascites. The second occurred in a 50-year-old woman who presented with pneumonia, hypoxemic respiratory failure, and a complicated parapneumonic effusion. *S. pseudoporcinus* was recovered from ascitic fluid and pleural fluid, respectively, and was identified using the VITEK 2 Compact (bioMérieux, Marcy-l’Étoile, France) system. Both patients received antimicrobial therapy and experienced favorable clinical outcomes without documented recurrence during follow-up. These cases expand the recognized clinical spectrum of invasive *S. pseudoporcinus* infection and emphasize the importance of accurate species-level identification of beta-hemolytic streptococci recovered from normally sterile sites.

## Introduction

Streptococci are Gram-positive, non-spore-forming, catalase-negative cocci that are typically arranged in pairs or chains. Most species are facultative anaerobes and can be classified according to their hemolytic patterns on blood agar and, for selected species, their Lancefield group antigens [1,2]. *Streptococcus agalactiae *(*S. agalactiae*), or group B *Streptococcus* (GBS), commonly colonizes the human gastrointestinal and genitourinary tracts but can cause disease in neonates, young infants, pregnant individuals, and older or immunocompromised adults. Invasive GBS disease is conventionally defined by the isolation of the organism from a normally sterile body site [3].

*Streptococcus pseudoporcinus* (*S. pseudoporcinus*) was first described in isolates obtained from the female genitourinary tract. These isolates had initially been identified biochemically as *Streptococcus porcinus* (*S. porcinus*); however, 16S rRNA gene sequencing demonstrated that the human isolates formed a distinct cluster with more than 2.1% sequence dissimilarity from *S. porcinus* strains of animal origin, leading to the proposal of *S. pseudoporcinus* as a novel species [4]. *S. pseudoporcinus* shares several biochemical and phenotypic characteristics with *S. agalactiae* and may cross-react with Lancefield group B antigen agglutination assays. Consequently, conventional phenotypic methods may misidentify *S. pseudoporcinus* as GBS, potentially leading to underrecognition of its prevalence and clinical significance [5,6].

In recent years, *S. pseudoporcinus* has been associated with a growing spectrum of human disease, including skin and soft-tissue infections, bacteremia, infective endocarditis, pneumonia with pleural infection, spontaneous bacterial peritonitis, osteomyelitis, endophthalmitis, and maternal sepsis with chorioamnionitis [5,7-20]. An earlier colonization study suggested that *S. pseudoporcinus* might be less frequently associated with invasive disease than *S. agalactiae*. However, subsequent reports of infections involving blood and other normally sterile sites have demonstrated its capacity to cause invasive and potentially life-threatening disease [6,7-20].

Here, we describe two cases of invasive *S. pseudoporcinus* infection presenting as spontaneous bacterial peritonitis and pneumonia with a complicated parapneumonic effusion, respectively, and review previously reported human infections caused by this emerging pathogen.

## Case presentation

This retrospective two-case series was conducted at Hospital General de San Juan del Río, located in San Juan del Río, Querétaro, Mexico. The first patient was treated in 2024 and the second in 2025, both within the Department of Internal Medicine. The cases were encountered consecutively within the service but were included as a convenience sample. Because no systematic search of institutional clinical or microbiology records was conducted to identify other potentially eligible cases, consecutive inclusion of all cases occurring at the institution during this period cannot be confirmed.

Case 1

A 59-year-old woman with biopsy-confirmed cirrhosis of undetermined etiology, diagnosed in 2014, presented to the emergency department with a three-week history of progressive abdominal distension, diffuse abdominal pain, nausea, jaundice, and worsening ascites. Previous evaluation had revealed no evidence of hepatitis B or C virus infection, significant alcohol consumption, diabetes mellitus, obesity, or autoimmune hepatitis. Her history was notable for previous episodes of ascites and upper gastrointestinal bleeding.

On admission, diagnostic and therapeutic paracentesis yielded 2,200 mL of turbid, milky-appearing ascitic fluid. Ascitic fluid and relevant serum laboratory findings are summarized in Table 1.

**Table 1:** Ascitic fluid and relevant serum laboratory findings in Case 1. Because universally accepted reference intervals are unavailable for several ascitic fluid parameters, established diagnostic or interpretive thresholds are provided where applicable.

Laboratory parameter	Result	Unit	Reference range or interpretive threshold
Ascitic fluid leukocyte count	410	Cells/mm^3^	0 – 5
Polymorphonuclear leukocytes	88	%	Not established reference range; absolute count is used diagnostically
Absolute polymorphonuclear leukocyte count	361	Cells/mm^3^	<250; >250 supports spontaneous bacterial peritonitis
Ascitic albumin	1.0	g/dL	Not established reference range
Serum albumin	3.99	g/dL	3.5 – 5.0
Serum-ascites albumin gradient	2.99	g/dL	>1.1 supports portal hypertension
Ascitic fluid total protein	2.3	g/dL	Not established reference range
Ascitic fluid glucose	88	mg/dL	Not established reference range
Ascitic fluid lactate dehydrogenase	118	U/L	Not established reference range
Ascitic fluid triglycerides	451	mg/dL	>200 supports chylous ascites

The absolute polymorphonuclear leukocyte count of 361 cells/mm³ supported the diagnosis of spontaneous bacterial peritonitis. A serum-ascites albumin gradient of 2.99 g/dL was consistent with portal hypertension, while the milky appearance and triglyceride concentration of 451 mg/dL supported chylous ascites.

A sample of ascitic fluid was submitted for microbiological culture. Empirical treatment with intravenous ceftriaxone, 2 g every 24 hours, was initiated. The culture yielded *S. pseudoporcinus*, identified using the VITEK 2 Compact system (bioMérieux, Marcy-l’Étoile, France). Antimicrobial susceptibility testing was not performed because appropriate testing for this uncommon organism was unavailable at our institution. Therefore, antimicrobial management was guided by the clinical response and susceptibility patterns previously reported in the literature. Blood cultures were not obtained.

During ceftriaxone therapy, the patient showed progressive clinical improvement, with resolution of abdominal pain and decreasing systemic inflammatory markers. Because clinically evident ascites persisted, a second therapeutic paracentesis was performed before the microbiological result from the initial ascitic fluid culture became available. The fluid remained turbid and milky-appearing, a finding attributed to the underlying chylous ascites in the context of clinical and inflammatory improvement; repeat cell count and biochemical analysis were not performed. Given the favorable clinical and inflammatory response, ceftriaxone was continued without modification. Abdominal computed tomography demonstrated ascites without evidence of perforation, abscess, or another intra-abdominal source of infection (Figure 1). 

**Figure 1:**
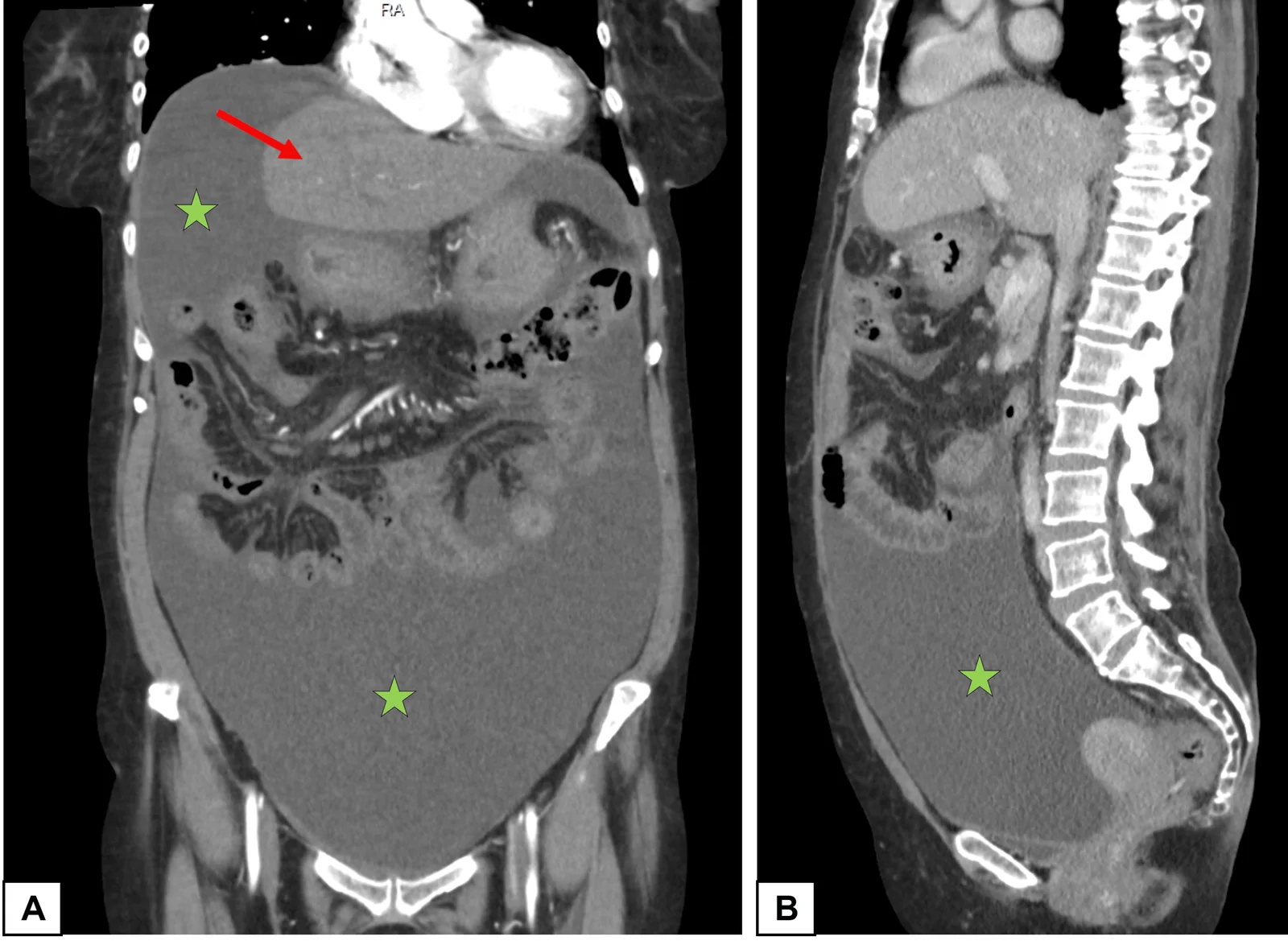
Contrast-enhanced abdominal computed tomography. (A) Arterial-phase coronal view and (B) venous-phase sagital view images demonstrate markedly reduced liver volume with cirrhotic morphology and large-volume ascites extending throughout the abdominal cavity, including the perihepatic spaces. In panel A, the red arrow indicates the liver; in both panels, green asterisks indicate ascitic fluid. No vascular or intestinal abnormalities or other intra-abdominal source of infection was identified.

Once the initial ascitic fluid culture yielded *S. pseudoporcinus*, intravenous ceftriaxone, 2 g every 24 hours, was continued to complete a 14-day course. The patient remained clinically stable, with resolution of abdominal pain and continued improvement in inflammatory markers. Culture of the ascitic fluid obtained during the second paracentesis showed no microbial growth. She was discharged on hospital day 14 with oral trimethoprim-sulfamethoxazole, 160/800 mg every 24 hours, prescribed indefinitely for secondary prophylaxis against spontaneous bacterial peritonitis. At the one-month outpatient follow-up, she remained clinically stable, with no clinically evident increase in ascites or other reported complications.

Case 2

A 50-year-old woman with a three-week history of cough, high-grade fever, and progressive dyspnea was referred to the emergency department. Her medical history included essential hypertension, major depressive disorder, and tobacco use disorder. On presentation, her respiratory rate was 24 breaths/min, heart rate 110 beats/min, oxygen saturation 85% on room air, body temperature 36.0°C, and blood pressure 127/85 mmHg. She was alert and oriented. Physical examination revealed decreased breath sounds over the right hemithorax, without jugular venous distention or peripheral edema.

Chest radiography and contrast-enhanced computed tomography demonstrated a right-sided pleural effusion with pleural thickening and the split pleura sign, raising suspicion for pleural infection (Figure 2). 

**Figure 2:**
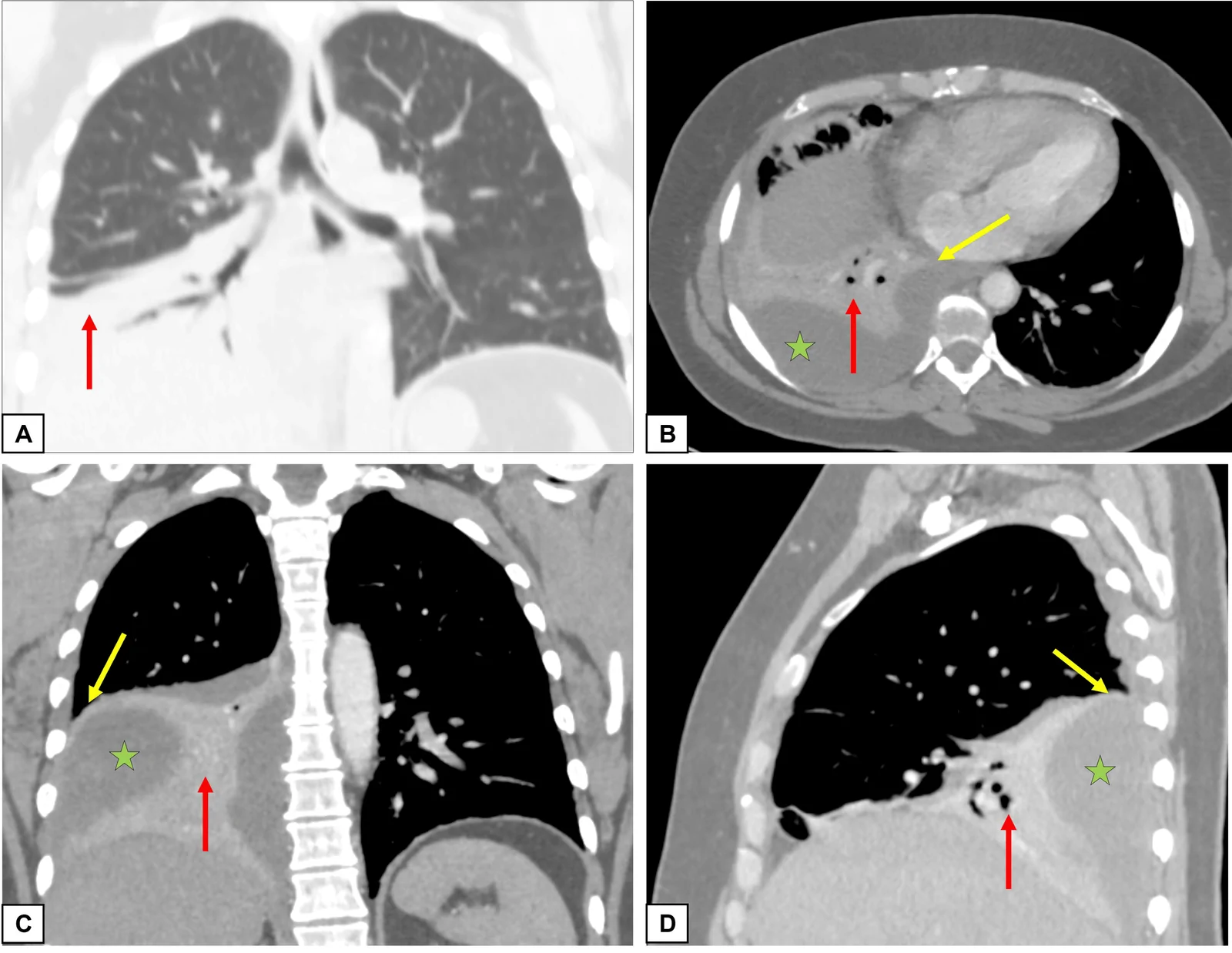
Noncontrast and contrast-enhanced chest computed tomography. (A) Lung-window image demonstrates pulmonary consolidation with air bronchograms, consistent with pneumonia. Axial (B), coronal (C), and sagittal (D) images demonstrate pulmonary consolidation, pleural effusion, and the split pleura sign, supporting the diagnosis of pleural infection. Red arrows indicate consolidation, the yellow arrow indicates the split pleura sign, the blue arrow indicates an air bronchogram, and green asterisks indicate pleural fluid.

Diagnostic thoracentesis was performed, and the pleural fluid was submitted for microbiological culture; no biochemical or cytological analysis was performed. Empirical antimicrobial therapy with intravenous ceftriaxone, 2 g every 24 hours, and oral clarithromycin, 500 mg every 12 hours, was initiated.

Pleural fluid culture yielded *S. pseudoporcinus*, identified using the VITEK 2 Compact system. Antimicrobial susceptibility testing was not performed because appropriate testing for this uncommon organism was unavailable at our institution. Consequently, therapeutic decisions were empirical and informed by the infection site, clinical evolution, and the limited susceptibility data available in the literature. Blood cultures were not obtained. Clarithromycin was administered for three days and discontinued after the culture result became available, whereas ceftriaxone was continued at 2 g intravenously every 24 hours to complete a seven-day course.

Despite seven days of ceftriaxone therapy, the patient had persistent dyspnea, leukocytosis, elevated C-reactive protein concentrations, supplemental oxygen requirements, and a persistent right-sided pleural effusion. No additional thoracentesis or pleural drainage catheter placement was performed. Because of the persistent clinical and inflammatory findings, antimicrobial therapy was changed to intravenous ceftazidime, 2 g every eight hours, for five days. The patient subsequently experienced progressive clinical improvement, with resolution of dyspnea, decreasing inflammatory markers, discontinuation of supplemental oxygen, and radiographic resolution of the pleural effusion. She was discharged on hospital day 12. At the two-month follow-up, she remained clinically stable without recurrent respiratory symptoms or other reported complications. Follow-up chest computed tomography performed six months after discharge demonstrated complete resolution of the pneumonia and pleural effusion (Figure 3).

**Figure 3:**
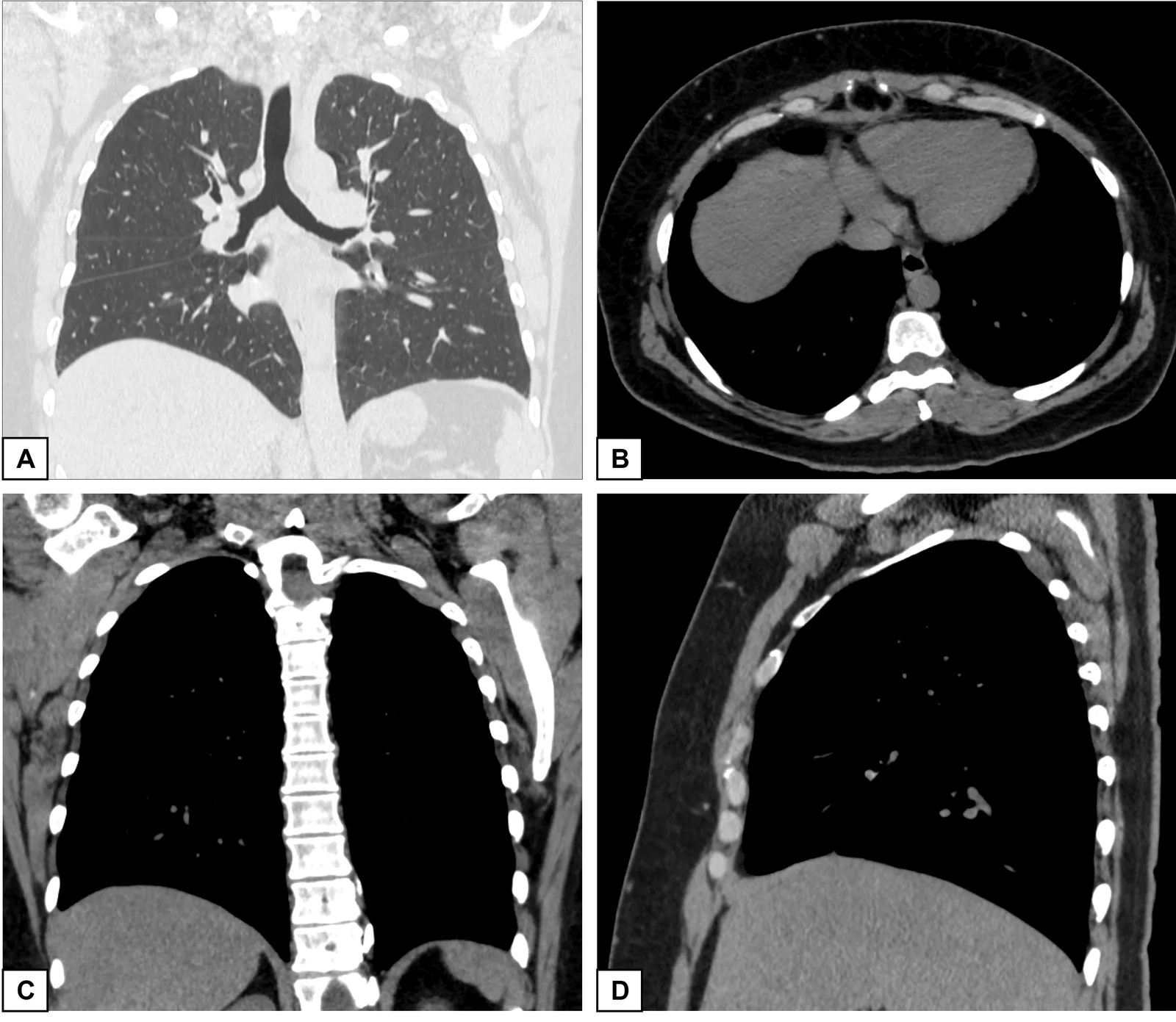
Follow-up noncontrast chest computed tomography performed six months after hospital discharge. Lung-window (A), axial (B), coronal (C), and sagittal (D) images demonstrate complete radiographic resolution of the pneumonia and pleural effusion, with no other thoracic abnormalities identified.

## Discussion

We describe two invasive infections caused by *S. pseudoporcinus*, presenting as spontaneous bacterial peritonitis in a patient with cirrhosis and as pneumonia with a complicated parapneumonic effusion in a second patient. In both cases, the organism was recovered from a normally sterile body fluid, supporting its pathogenic role rather than colonization. These cases add to the growing evidence that *S. pseudoporcinus* can cause clinically significant infections outside the female genitourinary tract.

Microbiological identification

*S. pseudoporcinus* is a Gram-positive, catalase-negative, nonmotile coccus typically arranged in chains. On blood agar, it produces small, smooth, beta-hemolytic colonies surrounded by a characteristically wide zone of complete hemolysis. Additional phenotypic characteristics include growth in 6.5% sodium chloride broth and variable growth at different temperatures. The isolates included in the original species description did not react with Lancefield group A, B, C, D, F, or G antisera [4]. Nevertheless, subsequent studies have demonstrated variable, reagent-dependent cross-reactivity between *S. pseudoporcinus* isolates and Lancefield group B antigen agglutination assays. Although *S. pseudoporcinus* does not possess the conventional group B antigen of *S. agalactiae*, shared or cross-reactive antigenic determinants among *S. porcinus*, *S. pseudoporcinus*, and *S. agalactiae* may produce positive group B reactions. Therefore, Lancefield serogrouping alone cannot reliably distinguish these species. Isolates that react with group B antisera but produce unusually wide zones of beta-hemolysis should raise suspicion for *S. pseudoporcinus* [21].

Automated systems such as VITEK 2 perform organism identification by analyzing biochemical reactions and can simultaneously conduct antimicrobial susceptibility testing. However, the phenotypic similarities between *S. pseudoporcinus* and *S. agalactiae* may complicate species-level identification using conventional microbiological methods. MALDI-TOF mass spectrometry provides rapid identification based on organism-specific protein spectral profiles and has been used to differentiate *S. pseudoporcinus* from GBS, provided that the reference database contains validated spectra for both organisms [6,22,23]. Sequencing of the 16S rRNA gene can serve as a reference method for identifying uncommon or phenotypically ambiguous bacterial isolates. This gene is widely used for bacterial identification because it contains both highly conserved and variable regions. Nevertheless, its discriminatory capacity may be limited for some closely related species, and sequence quality, database completeness, and similarity thresholds must be considered when interpreting results [24]. MALDI-TOF MS may provide faster and more accurate species-level identification than conventional automated biochemical systems like VITEK 2 [25].

In both of our cases, *S. pseudoporcinus* was identified using the VITEK 2 Compact system. Although this platform provided species-level identification, neither isolate was available for independent confirmation by MALDI-TOF mass spectrometry, 16S rRNA gene sequencing, or another molecular method. Consequently, the lack of confirmatory identification represents an important limitation of this case series. Although the lack of confirmatory identification precludes complete exclusion of misidentification, recovery of the organism from normally sterile body fluids in patients with compatible infectious syndromes supports the clinical significance of the isolates.

Spectrum of previously reported infections

A narrative literature search was conducted in PubMed during August 2026 using the search term “*Streptococcus pseudoporcinus* infection.” No publication-date restriction was applied. Titles and abstracts were screened to identify reports of human infection associated with *S. pseudoporcinus*. Full texts were reviewed when available, and cases involving clinical infection were included in the descriptive summary. When full-text access was unavailable, data were extracted from the abstract and other accessible bibliographic information. This search was not conducted as a systematic review and was not based on a prospectively registered protocol.

*S. pseudoporcinus* was initially isolated from the female genitourinary tract and was subsequently recognized as a colonizing organism in some women. Nevertheless, its recovery from a normally sterile body site should be considered clinically significant when accompanied by a compatible infectious syndrome [26]. Since its original description, *S. pseudoporcinus* has been associated with a broad spectrum of human disease, including skin and soft-tissue infections, bacteremia, infective endocarditis, maternal sepsis with chorioamnionitis, pneumonia with pleural infection, pyothorax, spondylodiscitis, and spontaneous bacterial peritonitis. Table 2 summarizes the clinical characteristics, microbiological findings, antimicrobial treatment, and outcomes of 16 previously reported patients and the two patients described in this case series.

**Table 2:** Clinical characteristics, microbiological findings, treatment, and outcomes of reported human Streptococcus pseudoporcinus infections. NA, not available in the accessible source; NR, not reported by the authors. For two reports, full texts were unavailable and data were extracted from the abstracts and accessible bibliographic sources.

Author	Year	Sex	Age	Relevant comorbidities or immunocompromising conditions	Clinical syndrome or primary site of infection	Site/specimen yielding S. pseudoporcinus	Bacteremia	Infection-related complications	Initial treatment	Subsequent antimicrobial therapy	Outcome
Mahlen & Clarridge [7]	2009	Male	33	NR	Soft tissue	Wound purulence	NR	NR	Cephalexin	None	Discharged
Sawamura et al. [8]	2018	Female	94	NR	Left lower leg cellulitis	Wound purulence	NR	Disseminated intravascular coagulation	Cefazolin	Meropenem & Vancomycin	Discharged
Pierce et al. [9]	2019	Female	41	NR	Chorioamnionitis	Urine, placenta, endometrium and blood culture	Yes	Stillbirth	Piperacillin-Tazobactam + Cefazolin Ampicillin + Gentamicin	Amoxicillin	Discharged against medical advice
Hai et al. [10]	2020	Male	40	NR	NR	Blood culture	Yes	Endocarditis	Cefoperazone-Sulbactam	Cefepime + Ofloxacin	Discharged
Gupta et al. [11]	2020	Male	43	- HIV infection - Syphilis infection	Genitourinary (suspected)	Blood culture	Yes	NR	Ceftriaxone	NR	NR
Khan et al. [12]	2020	Male	81	- Type 2 diabetes	Right lower leg cellulitis	Blood culture	Yes	- Acute respiratory failure - Endocarditis - Cardiac arrest	1. Ceftriaxone 2. Vancomycin	None	Death
		Female	72	NR	Community-acquired pneumonia	Lung tissue and pleural fluid	NR	- Complicated pleural effusion	1. Piperacillin-Tazobactam + Azithromycin 2. Ceftriaxone	Ertapenem	1. Outpatient parenteral microbial therapy unit 2. Discharged
Akagi et al. [13]	2020	Male	40	- Schizophrenia	Infective endocarditis	Blood culture	Yes	- Endocarditis	Vancomycin	Ampicillin-Sulbactam	Discharged
Vergadi et al. [14]	2021	Male	9	- Klippel-Trenaunay syndrome	Cellulitis	Blood culture	Yes	Cellulitis relapse	Ceftriaxone + Vancomycin	1. Vancomycin + Clindamycin 2. Trimethoprim-sulfamethoxazole	Discharged
Liatsos et al. [5]	2021	Male	56	- Cirrhosis - Type 2 diabetes - Coronary heart disease	Spontaneous bacterial peritonitis	Ascitic fluid and blood culture	Yes	- Septic shock - Acute liver failure	Meropenem + Daptomycin	None	Death
Otoni et al. [15]	2022	NA	NA	NA	NA	NA	NA	NA	NA	NA	NA
Venincasa et al. [16]	2022	Female	59	NA	Endophthalmitis	Vitreous culture	NA	Suspected globe perforation	Vancomycin + Ceftazidime	None	- Discharged - Permanent vision loss
Birlutiu et al. [17]	2023	Male	63	Mastocytosis	Infective endocarditis	Blood culture	Yes	- Endocarditis - Spondylodiscitis	Ceftriaxone + Vancomycin	Ceftriaxone + Levofloxacin	Discharged
Papapanagiotou et al. [18]	2023	Male	67	Diffuse large B-cell lymphomas	Left lower leg cellulitis	Blood culture	Yes	NR	Piperacillin-Tazobactam	Ceftriaxone	Discharged
Dong & Tian [19]	2023	Male	50	Chronic rhinitis	Orbital cellulitis	Orbital cavity pus	No	Corneal perforation	Ceftriaxone	Cefotaxime	- Discharged - Permanent vision loss
Chiko Y [20]	2025	Male	60	- Type 2 diabetes - Chronic pancreatitis	Community-acquired pneumonia	Pleural fluid and blood culture	Yes	- Pyothorax - Acute respiratory failure - Septic shock	Ampicillin-Sulbactam	Amoxicillin-Clavulanic acid	Discharged
Case 1	2026	Female	59	Cirrhosis of undetermined etiology	Spontaneous bacterial peritonitis	Ascitic fluid	Not assessed	NR	Ceftriaxone	None	Discharged
Case 2	2026	Female	50	- Essential hypertension - Tobacco use disorder	Community-acquired pneumonia and pleural effusion	Pleural fluid	Not assessed	Complicated parapneumonic effusion	Ceftriaxone + Clarithromycin	Ceftazidime	Discharged

Among the 18 patients included in the table, ten had documented bacteremia and several developed severe infection-related manifestations, including endocarditis, respiratory failure, disseminated intravascular coagulation, septic shock, acute liver failure, spondylodiscitis, and fetal death. Two patients died. These observations contrast with earlier suggestions that *S. pseudoporcinus *might be less frequently associated with invasive disease than *S. agalactiae* and support its potential to cause severe and occasionally fatal infection. However, because the available evidence consists predominantly of individual case reports, the frequency of invasive disease and its associated risk factors cannot be reliably estimated. Reported infections have occurred both in individuals without recognized immunocompromising conditions and in patients with diabetes mellitus, cirrhosis, human immunodeficiency virus infection, hematologic malignancy, or other relevant comorbidities. Therefore, the current literature is insufficient to determine whether immunosuppression constitutes an independent risk factor for *S. pseudoporcinus *infection.

Spontaneous bacterial peritonitis and chylous ascites

Our first patient presented with worsening ascites and abdominal pain. Ascitic fluid analysis showed 410 leukocytes/mm³, of which 88% were polymorphonuclear leukocytes, corresponding to an absolute polymorphonuclear leukocyte count of 361 cells/mm³. This value exceeded the diagnostic threshold of 250 cells/mm³ for spontaneous bacterial peritonitis. The serum-ascites albumin gradient was 2.99 g/dL, supporting portal hypertension as the mechanism underlying ascites formation. Additionally, the milky appearance of the fluid and an ascitic triglyceride concentration of 451 mg/dL were consistent with chylous ascites [27]. The patient had biopsy-confirmed cirrhosis of undetermined etiology and a history of previous decompensating events, including recurrent ascites and upper gastrointestinal bleeding. Cirrhosis may cause chylous ascites through increased hepatic lymph production and elevated lymphatic pressure, which can lead to dilatation and rupture of serosal lymphatic vessels. Chylous ascites is uncommon, accounting for approximately 0.5-1% of ascites associated with cirrhosis. In adults, its principal etiologies include malignancy, cirrhosis, and postoperative or traumatic disruption of the lymphatic system, while infections and congenital lymphatic disorders represent less frequent causes. An ascitic triglyceride concentration above 200 mg/dL is generally considered consistent with the diagnosis [28].

In our patient, ascitic fluid cytology did not reveal malignant cells, and abdominal computed tomography showed no intra-abdominal mass, lymphadenopathy, abscess, perforation, or other infectious source. She had no recent history of abdominal surgery or trauma. However, these findings did not definitively exclude every alternative etiology; the presence of cirrhosis, a serum-ascites albumin gradient consistent with portal hypertension, and the absence of another apparent cause made cirrhosis-associated chylous ascites the most plausible diagnosis. The positive monomicrobial culture, absolute polymorphonuclear leukocyte count above 250 cells/mm³, and absence of a surgically treatable intra-abdominal source supported spontaneous rather than secondary bacterial peritonitis. A previous case of spontaneous bacterial peritonitis caused by *S. pseudoporcinus* was reported in 2021 in a 56-year-old man with decompensated alcohol-associated cirrhosis, type 2 diabetes mellitus, and coronary artery disease. In that case, *S. pseudoporcinus* was recovered from both ascitic fluid and blood and was identified using the VITEK 2 system. Unlike our patient, the previously reported patient developed a rapidly progressive course characterized by septic shock, worsening hepatic dysfunction, severe acute kidney injury requiring hemodialysis, multiorgan failure, and death [5].

Both cases therefore demonstrate the ability of *S. pseudoporcinus* to cause spontaneous bacterial peritonitis in patients with cirrhosis, but their clinical courses differed substantially. Our patient experienced resolution of abdominal pain and a progressive decrease in systemic inflammatory markers during ceftriaxone therapy. The persistent milky appearance of the fluid obtained during the second therapeutic paracentesis was interpreted as a manifestation of the underlying chylous ascites rather than persistent infection. Ceftriaxone was consequently continued to complete a 14-day course, and the patient was discharged with a favorable short-term outcome.

Pneumonia and pleural infection

Our second patient presented with community-acquired pneumonia, hypoxemic respiratory failure, and a right-sided pleural effusion. Computed tomography demonstrated pulmonary consolidation with air bronchograms, pleural thickening, and the split pleura sign. Although pleural fluid pH, glucose, lactate dehydrogenase, protein concentration, and cell count were unavailable, recovery of *S. pseudoporcinus* from pleural fluid, together with the clinical and radiological findings, supported the diagnosis of pleural infection associated with a complicated parapneumonic effusion [29,30]. Persistent pleural effusion, supplemental oxygen requirements, and systemic inflammation despite initial antimicrobial therapy prompted modification of antimicrobial treatment from ceftriaxone to ceftazidime. No additional therapeutic pleural drainage procedure was performed after the initial diagnostic thoracentesis. The patient subsequently improved, with resolution of respiratory symptoms and pleural effusion, decreasing inflammatory markers, and discontinuation of supplemental oxygen. However, because isolate-specific antimicrobial susceptibility testing was unavailable, the clinical response cannot establish the activity or comparative efficacy of either cephalosporin against *S. pseudoporcinus*. The absence of pleural biochemical analysis also prevented classification based on pleural fluid pH or glucose, and the absence of a repeat culture precluded microbiological documentation of sterilization.

Our literature review identified two previously reported pleuropulmonary infections associated with isolation of *S. pseudoporcinus*. In 2020, a 72-year-old woman without major reported comorbidities presented with community-acquired pneumonia and subsequently developed a large, multiloculated left-sided pleural effusion with left upper lobe collapse. Empirical treatment with piperacillin-tazobactam and azithromycin was initiated [12]. She underwent video-assisted thoracoscopic surgery with decortication, and operative specimens yielded *S. pseudoporcinus* and *Prevotella oris*, both identified using the VITEK 2 system. Antimicrobial therapy was changed to ertapenem to provide coverage for both organisms, and the patient was discharged to complete outpatient parenteral antimicrobial therapy [12]. In 2025, a 60-year-old man with type 2 diabetes mellitus and a one-month history of cough and left-sided chest pain presented with shock, hypoxemic respiratory failure, and elevated inflammatory markers [20]. Computed tomography demonstrated a massive left-sided pleural effusion with pleural thickening and suspected intrapleural gas. The patient required invasive mechanical ventilation, intensive care, vasopressor support, and chest-tube drainage, which yielded a large volume of turbid, purulent-appearing fluid. *S. pseudoporcinus* and *Prevotella buccae* were recovered from pleural fluid. Treatment with intravenous ampicillin-sulbactam was continued, and the patient progressively improved; norepinephrine was discontinued on hospital day 4, he was extubated on day 8, the chest tube was removed on day 21, and he was discharged on day 29 after transition to oral amoxicillin-clavulanate [20].

Including our patient, three pleuropulmonary infections associated with *S. pseudoporcinus* have been described. The two previously reported pleuropulmonary infections required invasive pleural source control: video-assisted thoracoscopic surgery with decortication in the 2020 case and chest-tube drainage in the 2025 case. In contrast, no therapeutic pleural drainage procedure was performed in our patient after the initial diagnostic thoracentesis, and clinical and radiographic resolution occurred during antimicrobial treatment. The two previously published infections were polymicrobial and included *Prevotella *species, whereas *S. pseudoporcinus* was the only organism recovered from the pleural fluid in our case. This distinction strengthens its probable pathogenic role in our patient, although the lack of blood cultures, pleural biochemical analysis, and confirmatory species identification remains limiting. Given the limited number of reported cases and the polymicrobial nature of the two previous infections, no organism-specific conclusions regarding the need for pleural drainage can be drawn.

Antimicrobial management

Antimicrobial susceptibility testing was not performed because appropriate testing for this uncommon organism was unavailable at our institution. Consequently, antimicrobial selection was based on the infection site, clinical evolution, and susceptibility patterns previously reported in the literature rather than on isolate-specific susceptibility results. Both patients initially received ceftriaxone. The first patient showed a favorable clinical and inflammatory response without modification of antimicrobial therapy and completed a 14-day course. At discharge, trimethoprim-sulfamethoxazole was prescribed for secondary prophylaxis against spontaneous bacterial peritonitis. The second patient received intravenous ceftriaxone for seven days. Oral clarithromycin, 500 mg every 12 hours, was administered concomitantly during the first three days and discontinued after the pleural fluid culture result became available. Because of persistent respiratory and inflammatory findings, treatment was subsequently changed to intravenous ceftazidime, 2 g every eight hours, for five days. She subsequently experienced clinical, inflammatory, and radiographic improvement. However, because isolate-specific antimicrobial susceptibility testing was unavailable, neither cephalosporin can be considered microbiologically confirmed directed therapy, and the clinical response does not establish their comparative efficacy against *S. pseudoporcinus*.

No definitive antimicrobial regimen has been established for human infection caused by *S. pseudoporcinus*. The available evidence is limited to case reports and small observational studies using heterogeneous identification and susceptibility-testing methods. Published isolates (Table 3) have generally demonstrated susceptibility to beta-lactams and vancomycin, although resistance to macrolides, clindamycin, tetracyclines, and, occasionally, multiple antimicrobial classes has been described. These aggregated findings cannot establish the susceptibility of an individual isolate. Accordingly, neither ceftriaxone nor ceftazidime can be considered microbiologically confirmed directed therapy in our patients. Their favorable outcomes support the adequacy of the overall management strategies but do not establish the isolate-specific activity or comparative efficacy of either cephalosporin. Consequently, antimicrobial selection should be guided by the site and severity of infection, adequate source control, and isolate-specific susceptibility testing whenever available.

**Table 3:** Identification methods and reported antimicrobial susceptibility profiles of human Streptococcus pseudoporcinus isolates. Minimum inhibitory concentration (MIC) values are expressed in µg/mL; NA, not available in the accessible source; NP, not performed; NR, not reported by the authors; MALDI-TOF MS, matrix-assisted laser desorption/ionization time-of-flight mass spectrometry. For the two present cases here, MIC values and susceptibility results for penicillin, ampicillin, ceftriaxone, and ceftazidime were unavailable. The individual macrolides and fluoroquinolones tested were not specified in the available laboratory reports.

Author	Year	Identification method	Susceptible	Intermediate	Resistant	MIC without categorical interpretation
Mahlen & Clarridge [7]	2009	- Lancefield grouping - VITEK 2 - 16S rRNA sequencing	Susceptible to all other antimicrobial agents tested	NR	- Tetracycline	NR
Sawamura et al. [8]	2018	VITEK 2	- Cefepime (0.5) - Meropenem (0.12) - Vancomycin (≤0.25)	NR	- Penicillin - Ampicillin - Ceftriaxone - Azithromycin - Clarithromycin - Levofloxacin - Clindamycin	NR
Pierce et al. [9]	2019	- Lancefield grouping - MALDI-TOF MS - 16S rRNA sequencing	- Ampicillin - Clindamycin - Erythromycin - Penicillin - Vancomycin	NR	NR	NR
Hai et al. [10]	2020	VITEK 2	- Cefepime - Amoxicillin-clavulanate - Ofloxacin - Vancomycin - Erythromycin - Linezolid - Clindamycin - Chloramphenicol - Ciprofloxacin	NR	NR	NR
Gupta et al. [11]	2020	VITEK 2	- Vancomycin - Linezolid - Penicillin - Trimethoprim-sulfamethoxazole - Ciprofloxacin	NR	NR	NR
Khan et al. [12]	2020	Case 1: VITEK 2	NR	NR	NR	NR
		Case 2: VITEK 2	NR	NR	- Tetracycline	NR
Akagi et al. [13]	2020	NR	NR	NR	NR	NR
Vergadi et al. [14]	2021	- Lancefield grouping - VITEK 2 - Unspecified molecular analysis	NR	NR	NR	NR
Liatsos et al. [5]	2021	VITEK 2	- Vancomycin (0.5) - Daptomycin (0.19) - Linezolid (≤2) - Clindamycin (≤0.25) - Levofloxacin (0.5) - Tetracycline (≤0.25) - Rifampicin - Chloramphenicol	NR	- Ampicillin (8) - Amoxicillin-clavulanate - Penicillin G (≥8) - Cefuroxime - Cefotaxime - Ceftriaxone (4) - Meropenem - Erythromycin	NR
Otoni et al. [15]	2022	NA	NA	NA	NA	NA
Venincasa et al. [16]	2022	NA	NA	NA	NA	NA
Birlutiu et al. [17]	2023	VITEK 2	- Ampicillin - Cefotaxime - Ceftriaxone - Clindamycin - Chloramphenicol - Erythromycin - Tetracycline - Vancomycin - Moxifloxacin - Levofloxacin - Linezolid - Tigecycline	- Penicillin	NR	NR
Papapanagiotou et al. [18]	2023	- VITEK 2 - MALDI-TOF MS	- Penicillin (0.023) - Ampicillin (0.032) - Ceftriaxone (0.032) - Meropenem (0.008) - Clindamycin (0.19) - Levofloxacin (0.38) - Linezolid (1) - Tedizolid (0.38) - Vancomycin (0.25) - Daptomycin (0.032) - Dalbavancin (0.008) - Tetracycline (0.38) - Chloramphenicol (3)	NR	- Erythromycin (8)	NR
Dong & Tian [19]	2023	- BD Phoenix^TM^ system - Unspecified molecular analysis	- Cefepime - Linezolid - Ampicillin - Cefotaxime - Penicillin - Vancomycin	NR	NR	NR
Chiko Y [20]	2025	Not reported	- Penicillin (≤0.03) - Ampicillin (≤0.12) - Cefotaxime (0.25) - Cefepime (0.5) - Azithromycin (≤0.12) - Levofloxacin (0.5) - Ceftriaxone (≤0.25) - Meropenem (≤0.25) - Vancomycin (0.5) - Erythromycin (≤0.12) - Clindamycin (≤0.25) - Trimethoprim-Sulfamethoxazole (≤10)	NR	NR	- Amoxicillin clavulanate (≤1) - Ampicillin/Sulbactam (≤0.12) - Minocycline (≤0.12) - Imipenem/Cilastatin (≤0.12)
Case 1	2026	VITEK 2	NP	NP	NP	NP
Case 2	2026	VITEK 2	NP	NP	NP	NP

Limitations

This retrospective two-case series has several limitations. The patients constituted a convenience sample, and no systematic search of institutional microbiology records was conducted; therefore, complete case ascertainment cannot be assured, and the findings cannot define the incidence, risk factors, optimal treatment, or prognosis of invasive *S. pseudoporcinus* infection.

Identification relied exclusively on the VITEK 2 Compact system without confirmation by matrix-assisted laser desorption/ionization time-of-flight mass spectrometry or molecular sequencing, and misidentification cannot be completely excluded. Antimicrobial susceptibility testing and blood cultures were unavailable in both cases, precluding confirmation of susceptibility to the administered cephalosporins and assessment of concomitant bacteremia. Repeat ascitic fluid cell counts were unavailable in Case 1, while pleural fluid biochemical and cellular analyses, drained volume, and repeat culture were unavailable in Case 2. Alternative causes of chylous ascites could not be completely excluded, and follow-up was limited.

Finally, the accompanying narrative review used a single PubMed search term and may have missed reports not indexed in PubMed or described using different terminology. The published evidence consisted mainly of heterogeneous case reports, and two reports were available only through abstracts and bibliographic sources. Incomplete reporting and publication bias therefore limit characterization of the clinical spectrum and antimicrobial susceptibility profile of this organism.

## Conclusions

*Streptococcus pseudoporcinus* should be recognized as a potential cause of invasive disease beyond the genitourinary tract. Its recovery from normally sterile sites, including ascitic and pleural fluid, should be considered clinically significant when supported by compatible clinical and laboratory findings. Greater awareness, improved access to reliable species-level identification and validated antimicrobial susceptibility testing methods, and the development of appropriate interpretive criteria are needed to clarify the true frequency, clinical spectrum, and susceptibility profile of this uncommon pathogen and to guide antimicrobial management.
